# Disruption of the nuclear p53-GAPDH complex protects against ischemia-induced neuronal damage

**DOI:** 10.1186/1756-6606-7-20

**Published:** 2014-03-27

**Authors:** Dongxu Zhai, Kyle Chin, Min Wang, Fang Liu

**Affiliations:** 1 Department of Neuroscience, Centre for Addiction and Mental Health, Clarke Division, 250 College Street, Toronto, Ontario M5T 1R8, Canada; 2 Department of Psychiatry, University of Toronto, Toronto, Ontario M5T 1R8, Canada

## Abstract

Glyceraldehyde 3-phosphate dehydrogenase (GAPDH) is conventionally considered a critical enzyme that involves in glycolysis for energy production. Recent previous studies have suggested that GAPDH is important in glutamate-induced neuronal excitotoxicity, while accumulated evidence also demonstrated that GAPDH nuclear translocation plays a critical role in cell death. However, the molecular mechanisms underlying this process remain largely unknown. In this study, we showed that GAPDH translocates to the nucleus in a Siah1-dependent manner upon glutamate stimulation. The nuclear GAPDH forms a protein complex with p53 and enhances p53 expression and phosphorylation. Disruption of the GAPDH-p53 interaction with an interfering peptide blocks glutamate-induced cell death and GAPDH-mediated up-regulation of p53 expression and phosphorylation. Furthermore, administration of the interfering peptide *in vivo* protects against ischemia-induced cell death in rats subjected to tMCAo. Our data suggest that the nuclear p53-GAPDH complex is important in regulating glutamate-mediated neuronal death and could serve as a potential therapeutic target for ischemic stroke treatment.

## Introduction

GAPDH is traditionally thought to be a critical enzyme for glycolysis, and therefore, an important protein in energy production. However, recent evidence suggests that GAPDH is also involved in apoptosis, as indicated by changes in GAPDH expression and subcellular localization during apoptosis [1-4]. Indeed, GAPDH is not restricted to the cytosol, but it is also found in the nucleus, plasma membrane and extracellular space. The subcellular localization of GAPDH may be important for the multifuntional role of GAPDH.

Membrane-associated GAPDH binds to tubulin, thereby regulating polymerization and bundling of microtubules near the cell membrane. This suggests that GAPDH is involved in the organization of subcellular organelles [5]. Furthermore, release of tubulin from membrane-associated GAPDH facilitates the fusion of vesicles to the plasma membrane [6]. Interestingly, GAPDH can also be secreted from cells, where it can associate with the cell surface and inhibit cell spreading [7]. Yamaji et al. reported that GAPDH is detected in conditioned medium of cultured cell lines such as Cos-7, HEK-293 and neuro-2a, as well as rat serum [7]. In the cytosol, RNA/GAPDH interactions enable GAPDH to regulate protein translation by controlling the rate of protein synthesis and altering the stability of mRNA [8,9]. Furthermore, GAPDH is essential for ER to Golgi transport through interactions with Rab2 GTPase and atypical protein kinase C ℓ/λ (aPKCℓ/λ), both involved in the early secretary pathway and vesicle formation [10-12]. In the nucleus, GAPDH acts as a DNA binding protein and a t-RNA transport protein, and is important for the transportation and maintenance of nucleic acid [13,14]. The uracil DNA glycosylase activity of GAPDH, together with its ability to bind to diadenosine tetraphosphate, implies that GAPDH is involved in DNA replication and repair [15].

Recently, accumulated evidence has suggested that GAPDH nuclear translocation is associated with cell toxicity triggered by various agents, including glutamate [16]. Furthermore, the S-nitrosylation of GAPDH upon nitric oxide (NO) stimulation can trigger the nuclear translocation of GAPDH [4]. Several proteins, such as GOSPEL [17], AIRE [18], SIRT1 [19], Mitochondrial uncoupling protein 2 (UCP2) [20] and CIB1 [21] can promote or suppress the nuclear translocation of GAPDH in various cell types. However, the mechanism by which GAPDH activates the cell death pathway in the nucleus remains largely unknown, despite several studies have suggested the involvement of p53, a cellular tumor suppresser [22,23].

In the present study, we hypothesize that GAPDH translocates to nucleus upon glutamate stimulation. Subsequently, nuclear GAPDH forms a complex with p53 that leads to the activation of p53-mediated cell death pathway. Finally, we also hypothesize that GAPDH nuclear translocation plays a role in ischemic stroke, and disrupting the interaction of p53 and GAPDH may be neuroprotective.

## Materials and methods

### Peptide synthesis

The peptides were synthesized by Biomatik Corporation (Cambridge, USA). To facilitate the intracellular delivery of the peptide, both the GAPDH_2–2–1–1_ peptide and scrambled GAPDH_2–2–1–1_ peptide were fused to the cell membrane transduction domain of the HIV-1 TAT protein [YGRKKRRQRRR [24]] as previously described [25]. We refer to them here as: TAT-GAPDH_2–2–1–1_ and TAT -GAPDH_2–2–1–1-SCRM._ The amino acid sequence for the TAT-GAPDH_2–2–1–1_ peptide was YGRKKRRQRRRIPELNGKLTGMAFRVPTANV, and for TAT-GAPDH_2–2–1–1-SCRM,_ YGRKKRRQRRRVGNTALTKPGVNRLFEAPMI. The peptide was purified by HPLC to at least 90% purity. The peptide was dissolved in saline, aliquoted prior to use, and stored at -80°C.

### GST fusion proteins and mini-genes

The GST fusion protein and mini-genes are made as previously described [26-29]. Briefly, cDNA fragments were amplified by using PCR with specific primers to construct GST-fusion proteins and mini-genes encoding truncated GAPDH. Except where specified, all 5’ and 3’ oligonucleotides incorporated BamH1 (GGATCC) and Xho1 sites (CTCGAG), respectively, to facilitate sub-cloning into the pcDNA3 vector (for mini-gene construction) or into pGEX-4 T3 vector (for GST-fusion protein construction). GST-fusion proteins were prepared from bacterial lysates with glutathione sepharose 4B beads as described by the manufacturer (Amersham). To confirm appropriate splice fusion and the absence of spurious PCR-generated nucleotide errors, all constructs were re-sequenced.

### Protein affinity purification, co-immunoprecipitation and western blots

Protein affinity purification, co-immunoprecipitation and Western blot analyses were performed as previously described [29-31]. For affinity purification experiments, solubilized tissue extracts (800 μg protein) were incubated with glutathione-sepharose beads (GE Healthcare) bound to the indicated GST-fusion proteins (50 μg) at room temperature for 1 hour. Beads were washed, boiled for 5 min in SDS sample buffer and subjected to SDS-PAGE. After transfer of proteins onto nitrocellulose, membranes were Western blotted with the antibody of interest. For co-immunoprecipitation, briefly, solubilized tissue extracts (800 μg of protein) were incubated with protein A/G agarose for 4 hours at 4°C, followed by the addition of 20ul of protein A/G agarose (Santa Cruz Biotechnology) and p53 (polyclonal, Abcam, 2 μg) for 12 hours. Pellets were washed, boiled for 5 min in SDS sample buffer and subjected to 10% SDS-PAGE. 50 μg of striatal tissue extracted protein was used as control in each experiment. For Western blot, protein extracts were subject to 10% SDS-PAGE. After electrophoresis, protein was transferred to nitrocellulose membrane and blocked using 5% non-fat milk, followed by antibody incubation overnight. After rinsing the membrane for 5 min × 6 times, blot was incubated with desired HRP conjugated secondary antibody (1:5,000-1:10,000) for 2 h and the blot is incubated with Clarity Western ECL Substrate (Bio-rad) and developed in the ChemiDoc MP system (Bio-rad). The following antibodies were used for immunoprecipitation and Western blots: GAPDH (polyclonal from Abcam, 1:1000; monoclonal from Chemicon, 1:500), α-tubulin (monoclonal, Sigma-Aldrich, 1:10000), Siah1 (polyclonal from Abcam, 1:1000) and LaminB1 (Zymed Laboratories, 1:1000), p53 (immunoprecipitation, polyclonal, Abcam, 1000; western blotting, monoclonal, Abcam, 1:1000).

### Quantification of AMPAR-mediated excitotoxicity

HEK-293 T cells transfected with GluR1/2 subunits were exposed to 300 μM glutamate/25 μM cyclothiazide at 37°C for 24 hour. Cells were allowed to recover for 24 hours at 37°C. To quantify AMPAR-mediated cell death, the culture medium was replaced by a solution containing 50 μg/ml propidium iodide (PI) (Invitrogen, Carlsbad, CA). After 30 minute incubation at 37°C, fluorescence intensity in each well was measured with a plate reader (Victor3; PerkinElmer, Waltham, MA). The fraction of dead cells was normalized to the cell toxicity that occurred in either the glutamate-treated cells or KA-treated neurons. Primary cortical neurons were exposed to 100 μM KA/25 μM cyclothiazide in the presence of NMDAR and Ca^2+^ channel antagonists (10 μM MK-801 and 2 μM nimodipine, respectively) at 37°C for 1 hour.

### Transient middle cerebral artery occlusion (tMCAo) methods

All animals were purchased from Charles River Laboratories, Wilmington, MA. All animal procedures were approved by the Animal Care Committee of Center for Addiction and Mental Health in compliance with the relevant guidelines and regulations of the Canadian Council on Animal Care. Sprague Dawley rats weighing from 300 g-325 g (~7-8 weeks) were used for tMCAo modeling. Transient focal cerebral ischemia (90 minutes) was induced by right intraluminal middle cerebral artery occlusion (MCAo) as described previously [25,32]. Briefly, rats were anesthetized using 2.5% isoflurane supplemented with 97.5% compressed air. tMCAo was achieved by introducing a 3–0 monofilament suture into the middle cerebral artery via the internal carotid artery. During ischemia, body temperature was maintained at 36.5-37.5°C with a rectal feedback controlled homeothermic blanket system and a heating lamp [25]. 24 hours after tMCAo onset, rats were deeply anesthetized and sacrificed. The brains were cut into 1-mm-thick coronal sections and stained with 0.05% 2, 3, 5-triphenyltetrazolium chloride for 30 minutes at 37°C, followed by overnight immersion in 10% formalin. The infarct zone was demarcated and analyzed using Image J software. 2 hours after tMCAo and 10 minutes before the animals were killed, two neurological tests were performed [33]. The postural reflex test was used to examine upper body posture [34] while the forelimb placing test was used for sensorimotor integration in forelimb response to visual, tactile, and proprioceptive stimuli [35]. Performance on these tests was used to grade neurological function on a scale of 0–12 (0, normal; 12, worst). In the treatment groups, a single injection of 4 μl 10 mM TAT-GAPDH_2–2–1–1_ or TAT-GAPDH_2–2–1–1_ Scrambled peptide was infused intracerebroventricularly (i.c.v.) (AP: -1.0 mm; LM: -1.4 mm; DV: -3.6 mm from Dura) 30 minutes after the onset of tMCAo, whereas the sham operation group underwent all the surgical procedures except for the insertion of a suture.

### Intracerebroventricular (ICV) peptide delivery

30 min after the termination of MCAo suture insertion, peptide was delivered into the brain to investigate the treatment effect. ICV procedure was performed as previously described [36]. Rats were anesthetized with 3% isoflurane and placed in a stereotaxic apparatus (David Kopf Instrument, Tujunga, USA). The top of the skull was shaved and swabbed with an antiseptic, then a midline frontal incision was made in the scalp and the skin was retracted bilaterally. Burr holes (2 mm) were drilled into the skull and injecting needles were inserted at the following coordinate: 0.8 mm posterior to the bregma, 1.5 mm lateral to the midline, and 4.5 mm ventral to the surface of the skull. Rats received 4 μl infusions of either TAT-GAPDH_2–2–1–1_ or TAT-GAPDH_2–2–1–1-SCRM_ at a rate of 0.35 μl min^-1^ into the lateral cerebral ventricular. The injection cannula was left in place for 1 min following the infusion. Afterwards, the needle was slowly moved out and bone wax was applied to block the hole. Then the skull muscle and skin were stitched and rats were placed back to the cage to recover.

### Tetrazolium chloride (TTC) staining and infarct area measurement

TTC staining was performed as previously described with some modifications [36]. Briefly, 24 hours, 3 days and 5 days after tMCAO onset, rats were deeply anesthetized and decapitated. The brains were dissected and cut into 1-mm-thick coronal sections and stained with 0.25% 2, 3, 5-triphenyltetrazolium chloride for 15–25 minutes at 37°C, followed by overnight immersion in 4% formalin. The infarct zone was demarcated and analyzed with Image J software (Bethesda, MD). Edema is adjusted and normalized to the contralateral hemisphere of the same slices.

### Fluoro-jade B staining

Fluoro-Jade B staining was performed as previously described [36]. Animal used here is a new set of animals that is different from the ones used in TTC staining. Briefly, frozen sections (25 μm) of rat brains were used. Tissue slices were first immersed in 1% sodium hydroxide in 80% alcohol for 5 min, followed by 2 min in 70% alcohol and 2 min in distilled water. The slides were then transferred to a solution of 0.06% potassium permanganate for 10 min. After rinsing by distilled water for 2 min, the slices were stained in 0.0004% of the staining solution. Then the slices were rinsed and mounted for confocal microscopy. An unbiased stereological analysis was used to count the Fluoro-Jade B-labeled (FJ^+^) neurons in the layer 3 of the cortex [37]. The brain region sectioned for the Fluoro-Jade B staining is around the injection site (approximately ±2 mm Bregma of the injections site). In this region, the effect of the treatment is expected to be maximized due to the higher concentration of the TAT-GAPDH_2–2–1–1_ peptide.

### Statistical Analysis

All data presented here are presented as Mean ± SEM unless otherwise stated. Data are analyzed by either unpaired t test or one way ANOVA as stated. Statistical difference was considered significant if *P* < 0.05.

## Results

### Glutamate induces GAPDH nuclear translocation in a Siah1-dependent manner

**Figure 1:**
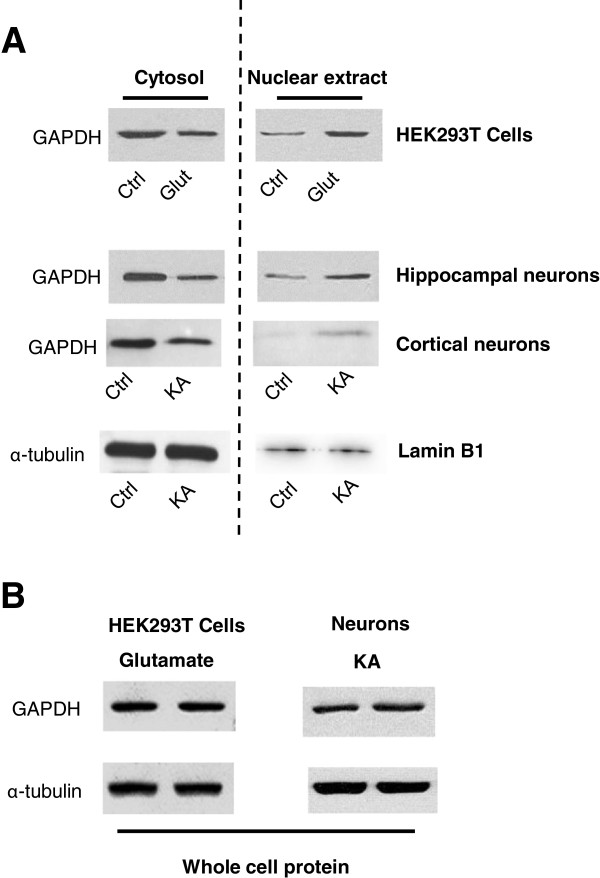
**The nuclear translocation of GAPDH is increased upon KA or glutamate treatment in neurons and GluR1/2 transfected HEK 293 T cells. A**. Representative lot of Western blot analysis of nuclear (right) and cytosol extract (left) using antibodies against GAPDH or α-tubulin in neurons or HEK 293 T cells upon KA or glutamate stimulation. **B**. Representative blot of Western blot analysis of total protein expression of GAPDH and α-tubulin in neurons or HEK 293 T cells upon KA or glutamate stimulation. All Western blot analysis was repeated for at least three times.

Previous studies have demonstrated that glutamate stimulation promotes GAPDH nuclear translocation, which leads to the activation of the cell death pathway [4]. Thus, we first confirmed whether GAPDH was translocated to the nucleus after agonist stimulation of AMPA receptors. We focused on AMPA receptors due to the tight association between AMPA receptor-mediated toxicity and ischemic neuronal death [38,39]. Consistent with previous reports, GAPDH expression in the nucleus was significantly increased following the activation of AMPA receptor in HEK-293 T cells expressing GluR1/2 subunits, and in rat primary cultured neurons from the hippocampus and cortex (Figure 1A). LaminB1 was used as the nuclear marker. There was no significant difference in the total expression of GAPDH between the control group and agonist stimulation group (Figure 1B).

**Figure 2:**
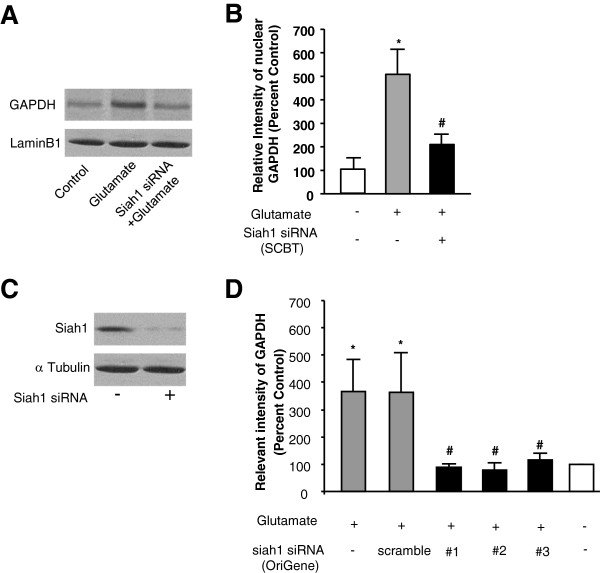
**The nuclear translocation of GAPDH upon GluR2 AMPA receptor activation depends on Siah-1. A**. Western blots verification of Siah 1 siRNA knockdown of the Siah-1 expression in HEK 293 T cells. **B**. Western blots analysis of GAPDH expression in the nucleus with/without glutamate treatment in the presence/absence of the Siah1 siRNA (Santa Cruz) in HEK-293 T cells expressing GluR1/2 subunits. LaminB1 was used as the nuclear marker. **C **&**D**, Quantification of nuclear expression of GAPDH with/without glutamate treatment in the presence/absence of the Siah1 siRNA (Santa Cruz), three Siah1 siRNA (#1, #2, #3, OriGene) and scrambled negative control siRNA (scramble, OriGene). *p < 0.05; significantly different from control group (n = 3 per group). #*p* < 0.05; significantly different from glutamate group (n = 3 per group). ANOVA, followed by *post-hoc* SNK test.

Sequence analysis of GAPDH revealed no typical nuclear localization signal (NLS). Previous studies have suggested that GAPDH translocates to nucleus through coupling with Siah1 [4,40], an E3 ubiquitin ligase with a NLS sequence [41]. Thus we tested whether Siah-1 is required for glutamate-mediated nuclear translocation of GAPDH by knocking down the expression of Siah 1 with siRNA (Santa Cruz Biotechnology). As shown in Figure 2A-B, Siah1 siRNA significantly inhibited glutamate-induced nuclear translocation of GAPDH. The Siah1 siRNA was highly effective in knocking-down the expression of Siah1, as shown in Figure 2C. To exclude off-target effects of the Siah1 siRNA, we tested an additional three Siah1 siRNAs and one scrambled negative control siRNA from ORIGENE (Figure 2D) for their ability to inhibit GAPDH nuclear translocation. Taken together, these data suggested that GAPDH may translocate into the nucleus in a Siah1 dependent manner.

### p53 forms a protein complex with GAPDH in the nucleus

**Figure 3:**
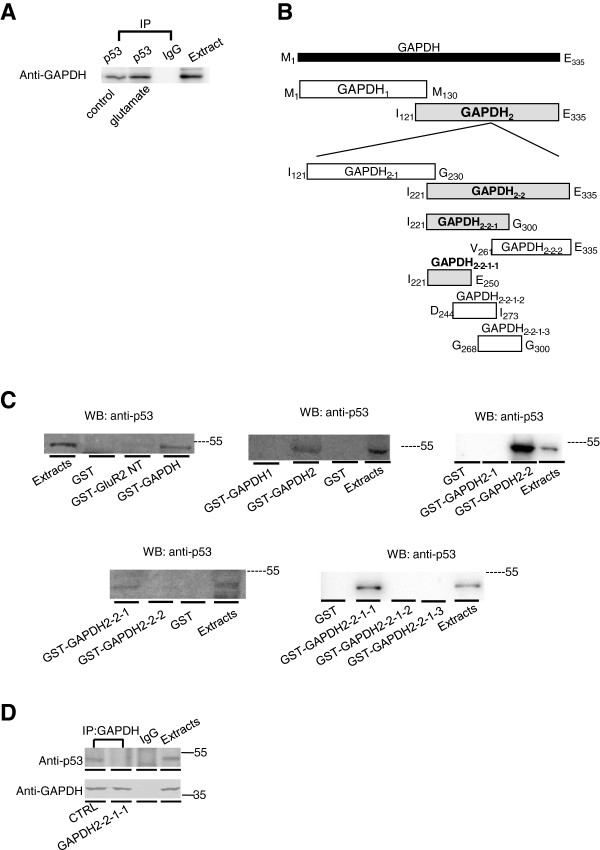
**Identification of GAPDH-p53 interaction fragment of GAPDH molecule. A**, CoIP assay of GAPDH/p53 using solubilized nuclear proteins extracted from rat hippocampal slices with/without glutamate treatment. **B**, Schematic representation of GST-fusion proteins encoding truncated GAPDH segments (gray colored bars indicate the positive fragments). **C**, Western blots of cortical nuclear p53 after affinity precipitation by different GST-GAPDH fragments (GST and GST-GAPDH, GST-GAPDH_1_ and GST-GAPDH_2_, GST- GAPDH_2–1_ and GST-GAPDH_2–2_, GAPDH_2–2–1_ and GAPDH_2–2–2_, GAPDH_2–2–1–1_, GAPDH_2–2–1–2_ and GAPDH_2–2–1–3_) **D**, CoIP of nuclear p53 by GAPDH primary antibody in HEK-293 T cells co-expressing GluR1/2 subunits with GAPDH_2–2–1–1_ peptide.

Several recent studies have shown that nuclear GAPDH forms a complex with p53 related protein [17,23]. p53, a tumor suppressor and transcription factor, has been implicated in glutamate-mediated excitotoxicity and ischemic neuronal damage [42-44]. Therefore we tested whether GAPDH forms a complex with p53 in the nucleus using co-immunoprecipitation (CoIP). As shown in Figure 3A, the p53 antibody was able to co-immunoprecipitate with GAPDH from solubilized nuclear proteins extracted from rat hippocampal slices. Interestingly, agonist stimulation facilitated p53-GAPDH complex formation, implicating that this protein complex is likely to be involved in glutamate-mediated function.

We then developed an interfering peptide that is able to disrupt the GAPDH-p53 complex to investigate the function of the p53-GAPDH interaction. We created the interfering peptide by identifying the amino-acid sequence of GAPDH that interacts with p53, through a series of affinity pull down experiments. Initially, we confirmed the interaction between p53 and GAPDH using a GST-fusion protein encoding GAPDH (GST-GAPDH). As shown in Figure 3C, GST-GAPDH, but not GST alone or GST-GluR2_NT_, can pull down p53 in rat hippocampal nuclear extracts. Furthermore, GST-fusion proteins encoding fragments of GAPDH were constructed and used in affinity purification assays (Figure 3B).

We concluded that the GAPDH: I_221_-E_250_ fragment facilitated the interaction with p53, since GST-GAPDH_2–2–1–1_ (I_221_-E_250_) was able to pull down p53 from solubilized nuclear protein extracts derived from rat hippocampus (Figure 3C). To confirm the critical role of the I_221_-E_250_ sequence in the GAPDH-p53 interaction, we tested whether a peptide encoding the I_221_-E_250_ region could block the GAPDH-p53 interaction using co-immunoprecipitation. As shown in Figure 3D, the ability of GAPDH antibody to co-immunoprecipitate with p53 was significantly blocked by pre-incubation with the I_221_-E_250_ peptide, indicating the I_221_-E_250_ region is essential in maintaining p53-GAPDH coupling.

### p53-GAPDH interaction plays an important role in AMPA receptor-mediated cell death

**Figure 4:**
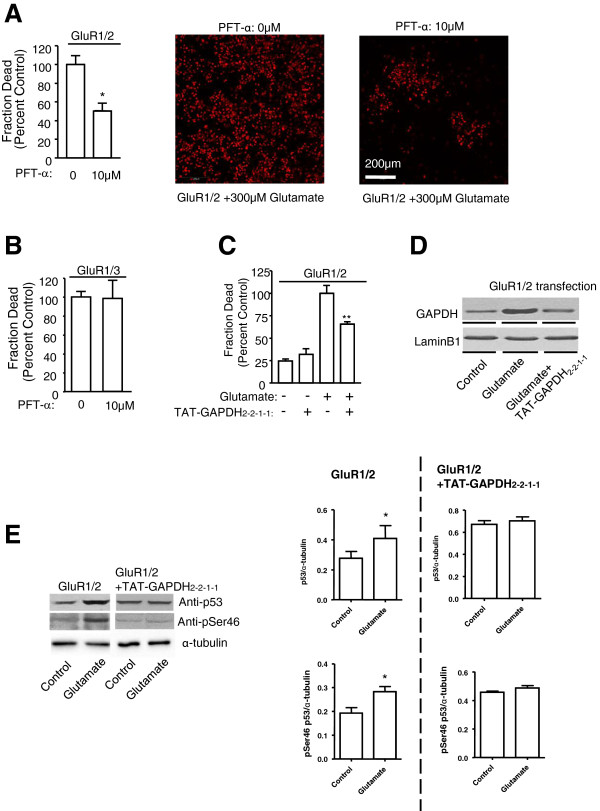
**p53-GAPDH interaction involves in GluR2 AMPA receptor mediated cell death. A**, Bar graph and representative figures summarizing the glutamate-induced cell death data in the presence/absence of p53 antagonist cyclic PFT-α (10 μM, 1 hr) in HEK-293 T cells expressing GluR1/2 subunits through quantitative measurements of PI fluorescence. *Significantly different from control group (*p* < 0.05; n = 9 per group); t-test. **B**, Bar graph summarizing the glutamate-induced cell death data in the presence/absence of p53 antagonist cyclic PFT-α in HEK-293 T cells expressing GluR1/3 subunits through quantitative measurements of PI fluorescence (n = 9 per group). **C**, Bar graph summarizing the glutamate-induced cell death data obtained from HEK-293 T cells co-expressing GluR1/2 subunits with/without the TAT-GAPDH_2–2–1–1_ peptide. **Significantly different from non-treatment/transfection group (*p* < 0.01; n = 9 per group); **D**. Western blots analysis of GAPDH expression in the nucleus with/without glutamate treatment in the presence/absence of TAT-GAPDH_2–2–1–1_ peptide in HEK-293 T cells expressing GluR1/2 subunits. LaminB1 was used as the nuclear marker. **E**, Western blots analysis of nuclear p53 and p53 phosphorylation at serine46 in HEK-293 T cells co-expressing GluR1/2 subunits with the GAPDH_2–2–1–1_ mini-gene upon glutamate stimulation. (t-test, *p < 0.05, n = 3).

We hypothesized that translocation of GAPDH to the nucleus following glutamate stimulation, may subsequently initiate cell death cascades through GAPDH-p53 complex formation. To test this hypothesis, we first confirmed whether p53 is involved in AMPA receptor-mediated excitotoxicity as previous reported [45]. As shown in Figure 4A, pre-treatment with the p53 inhibitor PFT-α (pifithrin-α, 10 μM, 1 hour) significantly inhibited glutamate-induced cell death in HEK-293 T cells expressing GluR1/2 subunits, whereas this effect was not seen in HEK-293 T cells expressing GluR1/3 subunits (Figure 4B). This result suggests that p53 may play a role in GluR2 containing AMPAR-mediated cell death.

We then hypothesized that if the p53/GAPDH complex is critical for AMPA receptor-mediated excitotoxicity, disruption of the p53-GAPDH interaction should inhibit AMPA receptor-mediated cell death. In HEK-293 T cells expressing GluR1/2, activation of AMPA receptor induces cell death. Pre-incubation with GAPDH_2–2–1–1_ peptide that disrupts the p53-GAPDH interaction inhibited AMPA receptor-mediated cell death (Figure 4C). To facilitate the intracellular delivery of the peptide, the GAPDH_2–2–1–1_ peptide was fused to the cell membrane transduction domain of the HIV-1 TAT protein [YGRKKRRQRRR [24]] to generate TAT-GAPDH_2–2–1–1_, as previously described [25]. Furthermore, we tested whether the TAT-GAPDH_2–2–1–1_ peptide would block the GAPDH nuclear translocation. As shown in Figure 4D, pre-incubation with TAT-GAPDH_2–2–1–1_ blocked glutamate-induced GAPDH nuclear translocation in HEK-293 T cells expressing GluR1/2 subunits.

Previous studies demonstrated a strong correlation between p53 expression and excitotoxic neuronal death [46-48], while other studies reported that phosphorylation can regulate p53 activity [49-52]. Thus, we tested whether AMPA receptor activation affected p53 expression or phosphorylation. As shown in Figure 4E, activation of AMPA receptors resulted in a 143 ± 16% (n = 3, p < 0.05) increase in p53 expression and a 147 ± 6% (n = 3, p < 0.05) increase in p53 phosphorylation in HEK-293 T cells expressing GluR1/2 subunits. These effects were blocked by the TAT-GAPDH_2–2–1–1_ peptide. Together, these data suggest that the p53-GAPDH interaction plays a critical role in AMPA receptor-mediated cell death.

### Disruption of the p53-GAPDH interaction protects against brain damage from focal ischemia in the rat

**Figure 5:**
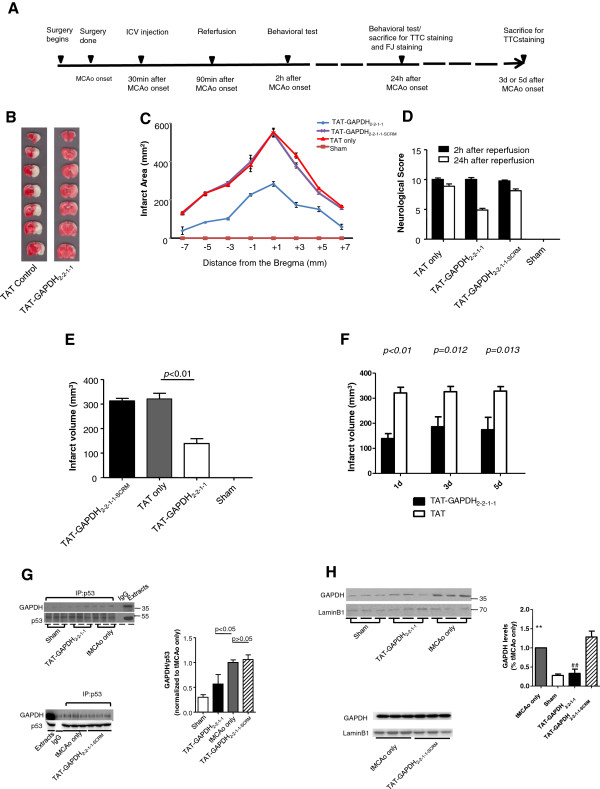
**TAT-GAPDH**_**2-2-1-1**_**peptide protects tMCAo model from neuronal damage. A**, schematic timeline of the transient ischemic stroke model and treatment. **B**, representative images of rat brain sections stained with TTC from rats subjected to tMCAo. TAT-GAPDH_2-2-1-1_ peptide (40 nmol) was administrated via i.c.v. injection 30 min after the onset of stroke. **C**, Infarct area of tMCAo rats brain in a series of brain slices according to the Stereotactic Coordinates in TAT-GAPDH_2-2-1-1_ and TAT only groups. **D**, neurological score of tMCAo rats in each group. Neurological score was assessed both 2 h and 24 h after reperfusion. ANOVA, followed by *post-hoc* SNK test (*p<0.05). **E**, Statistic analysis of the tMCAo infarct volume in each group. The infarct volume of TAT-GAPDH_2-2-1-1_ group is significantly smaller than that in the TAT only group (172.8±27.93 mm^3^*vs*. 296.0±41.11 mm^3^, n=8 in each group, *p*<0.05). ANOVA, followed by *post-hoc* SNK test. **F**, statistics analysis of tMCAo infarct volume after 1d, 3d and 5d of the stroke onsets (n=8 per group). **G**, CoIP of GAPDH by p53 from nuclear proteins in rat cortex of sham, TAT-GAPDH_2-2-1-1_ and tMCAo only groups (n=3 per group). ANOVA, followed by *post-hoc* SNK test. (two sets of experiments are normalized by the tMCAo only group) **H**, Western blot analysis of GAPDH nuclear expression in rat cortical tissues from sham, tMCAo, TAT-GAPDH_2-2-1-1_ (top) and TAT-GAPDH_2-2-1-1SCRM_ (bottom) groups. The experiments were conducted in two sets. The first set of experiments included three groups: sham, TAT-GAPDH2-2-1-1 and the tMCAo. The second set of experiments included two groups: the scrambled peptide and the tMCAo group. The bar graph represents each group normalized with the tMCAo group in the same set of experiments. **Significantly different from sham group (n=3 per group, *p*<0.01); ##significantly different from tMCAo group (n=3 per group, *p*<0.01); ANOVA, followed by *post-hoc* SNK test.

**Figure 6:**
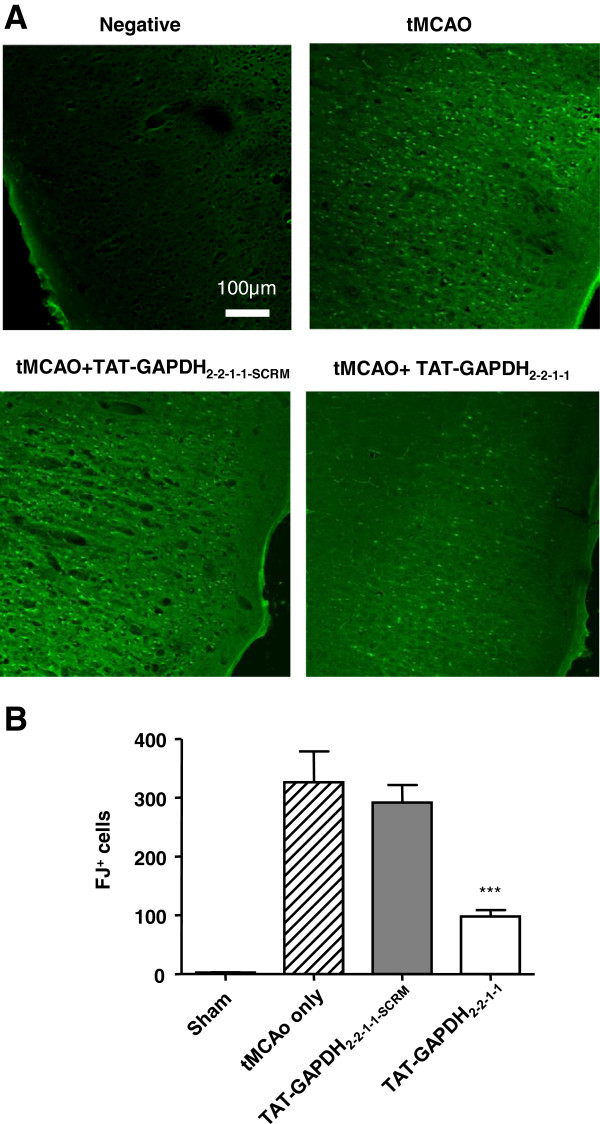
**Protective effects of the TAT-GAPDH**_**2–2–1–1**_**peptide against ischemic neuronal death. A**, Images of ischemic ipsilateral cortex from different treatment group of tMCAo rats were stained with Fluoro-Jade B 24 h after tMCAo onset. **B**, Bar graph shows summarized Fluoro-Jade B labeled cells (n = 5 per group, ***p < 0.001). Data are mean ± SEM, ANOVA followed by *post-hoc* SNK test.

Because AMPA receptor-mediated neurotoxicity has been implicated in excitotoxic neuronal death after ischemic stroke [53,54], we hypothesized that the p53/GAPDH complex might also play a role in ischemic brain damage. If so, an interfering peptide that is able to disrupt the p53-GAPDH interaction should reduce neuronal death following ischemia *in vivo*. To test this hypothesis, rat tMCAo modeling was achieved according to the schematic timeline shown by Figure 5A. An unbiased stereological analysis described previously [36] was used to count the FJ labeled neurons (FJ^+^) in the penumbra areas of the cortex. The cortex of the ischemic penumbra area is as previously defined [55-57]: from the edge of the infarction to 1 mm toward adjacent cerebral tissue. As shown in Figure 6A-B, the majority of the neurons in the tMCAo group (326.0 ± 52.73) and scrambled TAT-GAPDH_2–2–1–1_ peptide group (291.6 ± 29.77) had degenerated. However, significantly more neurons survived in the TAT-GAPDH_2–2–1–1_ treated group (97.8 ± 10.97, *p* < 0.001, compared with the tMCAo group and TAT-GAPDH_2–2–1–1-SCRM_ group). Furthermore, the infarct volume is significantly smaller in tMCAo rats treated with TAT-GAPDH_2–2–1–1_ peptide (172.8 ± 27.93 mm^3^*vs.* 296 ± 41.11 mm^3^, Figure 5B-C&E) 30 minutes after the tMCAo onset. Consistent with the histology, neurological function is significantly better with TAT-GAPDH_2–2–1–1_ peptide treatment (Figure 5D). In order to investigate the neuroprotective effect of this peptide, we have performed this model for a longer survival period. As shown in Figure 5F, the protective effect of our peptide can be observed 5 days post MCAo onset.

To confirm that the observed protective effect of TAT-GAPDH_2–2–1–1_ peptide was due to the disruption of p53-GAPDH interaction and the blockade of GAPDH nuclear translocation, we measured the p53-GAPDH interaction in tMCAo rats with co-immunoprecipitation. As shown in Figure 5G, the p53-GAPDH interaction is significantly increased in the ischemia group compared to the sham and the TAT-GAPDH_2–2–1–1_ peptide-treated groups. This increase was blocked by the TAT-GAPDH_2–2–1–1_ peptide administered 30 minutes after ischemia via stereotaxic i.c.v. injection. Similarly, the nuclear translocation of GAPDH was also enhanced in the ischemia group and the ischemia-induced enhancement was inhibited by the application of the TAT-GAPDH_2–2–1–1_ peptide (Figure 5H). These data together suggest that the p53-GAPDH interaction may be involved in the pathophysiology of ischemic stroke. Disruption of the p53-GAPDH interaction protects against ischemia-induced neuronal damage and increases survival after cerebral ischemia.

## Discussion

GAPDH nuclear translocation has been implicated in the pathogenesis of neuronal death [40,50,58,59]. However, the underlying mechanisms remain unclear. We have demonstrated in the current study that glutamate stimulation promotes GAPDH nuclear translocation in a Siah1-dependent manner. Upon entering the nucleus, GAPDH forms a complex with p53 and activates the p53-dependent cell death pathway. We also found an enhanced p53-GAPDH interaction in brains of rats subjected to tMCAo. We then used an interfering peptide to block the p53-GAPDH interaction and reduce brain damage following focal ischemia. These experiments suggest that the p53-GAPDH interaction plays an important role in the pathophysiology of ischemic stroke.

GAPDH is overexpressed and accumulates in the nucleus during apoptosis induced by a variety of insults. Evidence shows that the GAPDH nuclear translocation is essential for the apoptotic cascade [1,2]. Antisense oligonucleotides that deplete GAPDH prevent this nuclear translocation and reduce apoptosis [1,3,4]. The mechanism underlying GAPDH nuclear translocation and subsequent cell death remains largely unknown. However, recent studies have described several factors that may be involved: (1) the expression of GAPDH is regulated by p53, a tumor suppressor protein and proapoptotic transcription factor, which suggests that GAPDH could be a downstream apoptotic mediator [60]; (2) overexpression of Bcl-2 blocks the apoptotic insults triggered by GAPDH overexpression, nuclear translocation and subsequent apoptosis, suggesting that Bcl-2 may participate in the regulation of GAPDH nuclear translocation, consistent with the anti-apoptotic function of Bcl-2 [61]; (3) GAPDH binds to a nuclear localization signal-containing protein, Siah1 which initiates GAPDH translocation to the nucleus. The association with GAPDH stabilizes Siah1 and thereby enhances Siah1-mediated proteolytic cleavage of its nuclear substrates, triggering apoptosis [4,40,58,62]. We have shown that the knock-down of Siah1 expression inhibits GAPDH nuclear translocation, providing further support for the idea that glutamate-induced GAPDH nuclear translocation is dependent on Siah1.

Consistent with previous studies, our data suggest that glutamate-induced cell death and ischemic neuronal damage are associated with the nuclear translocation of GAPDH and the formation of a p53-GAPDH complex in the nucleus. Because we observed GAPDH nuclear translocation in several cell culture types (HEK-293 T expressing GluR1/2 subunits treated with glutamate, rat hippocampal and cortical primary cultures treated with KA), the p53-GAPDH interaction could be a more general mechanism for cell toxicity. Of course more work is needed to confirm the exact role of p53-GAPDH interaction in cell toxicity.

We identified the sequence of GAPDH (I_221_-E_250_) that couples with p53. Hara et al. [4] reported that GAPDH could exert apoptotic effects through binding to siah-1 for nuclear translocation. They reported the 220–233 regions as the interacting site enabling GAPDH to couple with Siah1, which is within the GAPDH-p53 interacting site. Although further experiments are required to identify the exact amino acid sequences for complex formation in the two proteins, we speculate that p53 may compete with Siah1 to bind with GAPDH once the Siah1-GAPDH complex enters the nucleus. Thus, activation of AMPA receptor may lead to a sequential protein-protein interaction that begins with GAPDH-Siah1 complex nuclear translocation followed by the uncoupling of GAPDH-Siah1 and GAPDH-p53 complex formation that eventually activates the p53-dependent cell death pathway.

Almost any DNA-damaging agent can cause apoptosis of neurons, and this apoptosis is dependent upon p53 [42]. The first indication that p53 is important for neuronal damage following ischemia or excitotoxicity came from studies showing that p53 levels are increased in response to these insults [42]. Of particular interest are studies demonstrating that: (1) neuronal death in the hippocampal CA1 region was much more extensive in p53^+/+^ than in p53^-/-^ mice subjected to transient global ischemia [63], and (2) transient focal ischemia induced by tMCAo led to significant ischemic damage in p53^+/+^ but not in p53^+/-^ mice [64]. The molecular mechanisms by which p53 is activated and accumulates under conditions of cellular stress may include either phosphorylation or acetylation of p53 [65].

In our *in vivo* study, we did not observe a treatment effect when peptide is administrated 1 h after the stroke onset (data not shown). This may indicate a limited time window for the effectiveness of our peptide treatment. However, this limit may be because of the modeling system chosen--we did observed a delayed protective effect using another peptide on rodent global ischemia model [36], which seems due to the different modeling system from the suture insertion tMCAo model. Only when we observe the delayed treatment effect (administrated 1 h or later after stroke onset) with our TAT-GAPDH_2–2–1–1_ peptide in the future experiments, we can confirm the delayed effect is indeed because of the modeling issue. Furthermore, intracranial peptide delivery, although at 30 min after termination of suture-insertion surgery, may affect cerebral blood flow (particularly for the reversible MCAO) and/or other factors and thus may have neuro-protective actions independent of the mechanisms proposed for the peptide treatment. Although proper control group is set up, we cannot totally exclude this confound factor that may affect the outcome of the peptide treatment. Also, we found a trend of higher survival of tMCAo rats with TAT-GAPDH_2–2–1–1_ peptide treatment. Although larger sample size is required to secure a valid statistical analysis, only ~56% (9 out of 16) of tMCAo rats survived with the control peptide, while ~92% (11 out of 12) of tMCAo rats survived with the TAT-GAPDH_2–2–1–1_ peptide.

In the present study, the p53 inhibitor PFT-α prevented glutamate-induced cell death, suggesting the involvement of p53 in AMPA receptor-mediated cell death. Moreover, p53 couples to GAPDH and the disruption of this interaction significantly inhibits glutamate-induced cell death and ischemia induced neuronal damage. Our data may provide an opportunity to develop novel therapeutic agents for the treatment of ischemic stroke.

## Competing interests

The authors declare that they have no competing interests.

## Authors’ contributions

DXZ and MW carried out the experiments and the manuscript preparation, KQ participated in the animal studies, FL supervised the study and wrote the manuscript. All authors read and approved the final manuscript.
